# Prefrontally modulated vagal neuroimmunomodulation is associated with telomere length

**DOI:** 10.3389/fnins.2022.1063162

**Published:** 2022-12-20

**Authors:** Torvald F. Ask, Stefan Sütterlin

**Affiliations:** ^1^Faculty of Health, Welfare and Organisation, Østfold University College, Halden, Norway; ^2^Department of Information Security and Communication Technology, Norwegian University of Science and Technology, Gjøvik, Norway; ^3^Faculty of Computer Science, Albstadt-Sigmaringen University, Sigmaringen, Germany

**Keywords:** inflammation, c reactive protein (CRP), telomere length, the neuro-immuno-senescence integrative model, vagal neuroimmunomodulation, cellular senescence, vagal tone, prefrontal cortex-autonomic nervous system-spleen axis

## Abstract

**Background:**

Accumulated senescent cells are proposed to be one of the main drivers of age-related pathology such as dementia and cancer through disruption of tissue structure and function. We recently proposed the Neuro-Immuno-Senescence Integrative Model (NISIM), which relates prefrontally modulated vagal tone and subsequent balance between vagal and sympathetic input to the spleen to inflammatory responses leading to generation of reactive oxygen species and oxidative telomere damage.

**Aim:**

In this study, we assess inflammation as a mediator in the relationship between prefrontally modulated vagal tone and leukocyte telomere length (LTL). We also assess the relationship between a recently proposed index of vagal neuroimmunomodulation (vagal tone/inflammation ratio; NIM index) and telomere length.

**Methods:**

This study uses participant data from a large nationally representative longitudinal study since 1974 with a total of 45,000 Norwegian residents so far. A sub-sample of 131 participants from which ultrashort recordings (30 s) of vagal tone, c reactive protein, and LTL could be obtained were included in the study. Relationships were analyzed with Pearson’s correlations and hierarchical multiple linear regression using either vagal tone and CRP or the NIM index to predict telomere length.

**Results:**

Vagal tone was a significant positive predictor of telomere length but this was not mediated by c reactive protein, even after controlling for confounders. The NIM index was a significant positive predictor of telomere length, also when controlling for confounders. In a follow-up analysis simultaneously comparing telomere length between groups with high and low values of vagal tone, and between groups with high and low NIM index values, telomere length was only significantly different between NIM index groups.

**Conclusion:**

This is the first study suggesting that prefrontally modulated vagal neuroimmunomodulation is associated with telomere length thus supporting the NISIM. Results indicate that the NIM index is a more sensitive indicator of vagal neuroimmunomodulation than vagal tone and CRP in isolation.

## 1 Introduction

In this paper, we test the previously postulated hypothesis that telomere length is influenced by prefrontally modulated vagal neuroimmunomodulation (Ask et al., 2018) to elucidate the mechanisms linking individual differences in psychophysiological stress regulation capacity to aging and age-related pathophysiology. We do this by assessing whether inflammation mediates the relationship between vagally mediated heart rate variability (vmHRV) and telomere length. We also test whether a recently proposed index for vagal neuroimmunomodulation (Gidron et al., 2018) is associated with telomere length. The shift toward considering aging as a disease has prompted increasing efforts to understand its many underlying mechanisms and their subsequent role in degenerating pathologies (e.g., Campisi, 2000, 2013; Kane and Sinclair, 2019). These efforts converge on the ambition that if humans can fully understand the physiological drivers of aging then perhaps medical- and behavioral interventions can be developed such that age-related pathologies can be eradicated altogether.

Cellular senescence, often referred to as cellular aging, is hypothesized to be one of the main culprits of age-related pathology and is induced by cellular stressors such as genomic instability, telomere attrition, and mitochondrial dysfunction (Campisi, 2000, 2013; Rossiello et al., 2022). Following DNA damage, a network cascade of proteins is activated as part of the DNA damage response, leading to the phosphorylation of P53. Whether DDR activation will result in cellular senescence or another response such as DNA repair or apoptosis depends on the nature and persistence of the DNA damage response-signal and the cellular stressor (Campisi, 2000, 2013; Wang et al., 2021). Due to being resistant to apoptosis, senescent cells accumulate with age. Some senescent cells start secreting a wide range of ligands including pro-inflammatory molecules and growth factors that over time promote disruption in tissue structure and function, thus altering intercellular communication (Campisi, 2000, 2013; Tchkonia et al., 2013). This is known as the senescence-associated secretory phenotype and is a suggested driver of age-related pathologies such as neurodegenerative diseases (Baker et al., 2011; Chinta et al., 2015) and cancer (Bissell et al., 2005; Rao and Jackson, 2016). Individual anato-physiological factors driving the antecedents to cellular senescence are still poorly understood.

**FIGURE 1:**
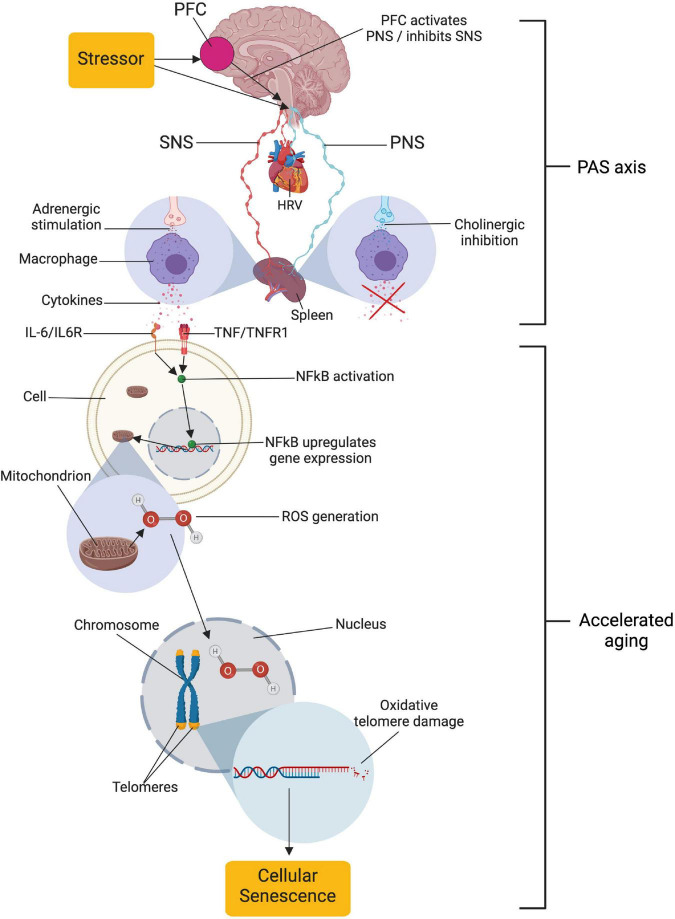
Relationships proposed by the neuro-immuno-senescence integrative model (NISIM). The NISIM propose that the link between psychological stress and telomere length starts with (1) prefrontal capacity for increasing activity in the vagus nerve, (2) vagal input to the spleen modulating the inflammatory response, (3) subsequent inflammatory load increasing Nuclear Factor Kappa B (NFkB) activity and reactive oxygen species generation, and (4) the resulting oxidative damage to telomeres (Ask et al., 2018). PFC, prefrontal cortex; SNS, sympathetic nervous system; PNS, parasympathetic nervous system; HRV, heart rate variability; PAS axis, prefrontal cortex-autonomic nervous system-spleen axis; IL-6, interleukin 6; IL6R, IL6 receptor; TNF, tumor necrosis factor; TNFR1, TNF receptor 1; ROS, reactive oxygen species; HOOH, hydrogen peroxide. Created with BioRender.com.

In a seminal paper, Epel et al. (2004) made the pioneering discovery that psychological stress is related to shorter telomere length, an antecedent to cellular senescence (Campisi, 2000, 2013; Coluzzi et al., 2019). While being a critical contribution to our understanding of how psychological stress relates to ill-health, the specific mechanisms relating these phenomena have remained rather elusive for the remaining decade-plus despite considerable scientific inquiry (e.g., Epel et al., 2009; Morey et al., 2015). To bridge this gap, we recently proposed a model (Figure 1) outlining specific anato-physiological mechanisms relating psychological stress regulation to cellular senescence, and subsequently, age-related pathology (Ask et al., 2018). The model is largely based on the prefrontal component of the central-autonomic network, the immuno reflex (or cholinergic anti-inflammatory pathway; Tracey, 2002), and the molecular cell biology related to generation of reactive oxygen species (e.g., Schreck and Baeuerle, 1994) and oxidative telomere damage (Brand, 2019; Coluzzi et al., 2019; Wang et al., 2021).

In the model, termed the Neuro-Immuno-Senescence Integrative Model (NISIM; Ask et al., 2018), the prefrontal capacity for modulation of vagal activation, quantified as vmHRV (Brunoni et al., 2013; Nikolin et al., 2017) during exposure to psychological stress (Hildebrandt et al., 2016; Chand et al., 2020), consequently affect the balance of vagal and sympathetic input to the spleen (Tracey, 2002). Low capacity for prefrontal vagal modulation translates to low vmHRV (Thayer and Lane, 2000), indicating reduced vagal input to the spleen and increased inflammation (Williams et al., 2019). Increased inflammatory load is negatively associated with telomere length (Fitzpatrick et al., 2007; O’Donovan et al., 2011; Masi et al., 2012; Wong et al., 2014), while indicators of vagal tone such as respiratory sinus arrhythmia (Kroenke et al., 2011) and vmHRV is positively associated with telomere length (Zalli et al., 2014; Perseguini et al., 2015; Streltsova et al., 2017; Woody et al., 2017; Wilson et al., 2019; Shahane et al., 2020). Together, the outlined above mechanisms are how the NISIM relates psychological stress regulation capacity to cellular senescence in a dynamic framework that accounts for some of the inter-individual differences in the expression of age-related phenotypes (Ask et al., 2018).

It is important to note that the mechanisms outlined by the NISIM are not suggested to be the main drivers of cellular senescence. The NISIM should rather be viewed as detailing the neuroimmunological mechanisms underlying individual differences in small but cumulative antecedents to cellular senescence that accelerate the transition from health to age-related pathology (Ask et al., 2018). Because the NISIM argues that the PFC affects splenogenic inflammation levels when exerting modulatory influences on arousal *via* the vagus nerve (Brunoni et al., 2013; Nikolin et al., 2017; Chand et al., 2020), the neural pathways facilitating these relationships can be conceptualized as a PFC-autonomic nervous system (ANS)-Spleen (PAS) axis. Inability of the PFC to exert modulatory influences *via* the PAS axis during stress exposure results in cumulative telomere attrition (Ask et al., 2018). Thus, according to the NISIM, the PAS axis is the common denominator in both how individual differences in stress regulation capacity translates to inflammation-related pathology as well as explaining why they are subtle and accumulate over time.

Both shorter telomeres and increased peripheral levels of inflammation are associated with increased risk for Alzheimer’s disease (King et al., 2018; Wood, 2018; Hackenhaar et al., 2021). Whether shorter telomeres are a risk for cancer development might depend on cancer type (Zhu et al., 2016), although damage-induced telomere dysfunction can occur irrespective of telomere length (Victorelli and Passos, 2017; Brand, 2019). A series of studies associating vagal tone with cancer survival provided some of the first evidence suggesting that vagal modulation of the immune system could be a manner of life and death in clinical conditions (Mouton et al., 2010, 2012; Chiang et al., 2013; De Couck et al., 2013). In absence of a biomarker serving as index for this relationship, Gidron et al. (2018) proposed the vagal neuroimmunomodulation (NIM) index; a simple numerical ratio (vmHRV/CRP) indexing vagal immunomodulation of inflammation. Higher values on the NIM index have been shown to be a protective factor against cancer (Gidron et al., 2018), while lower values are associated with all-cause mortality (Jarczok et al., 2021). Inflammatory markers do not necessarily reflect vagal activity, nor does vagal activity necessarily reflect inflammation. Thus, the NIM index could potentially be a more sensitive measure of PAS axis activity than any of the measures in isolation. How NIM index scores, and by extent PAS axis activity, relate to antecedents of cellular senescence such as telomere length has yet to be explored.

The NISIM (Ask et al., 2018) proposes that for psychological effects on telomere length, prefrontal cortical influences on the vagus nerve modulate the inflammatory response with reduced telomere length being downstream of inflammation. Thus, the main aim of this paper is to test the hypothesis that inflammation negatively mediates the relationship between vagal tone and telomere length. The secondary aim is to assess whether the NIM index (Gidron et al., 2018) could serve as a useful index for the PAS axis in the NISIM by assessing whether the NIM index is a positive predictor of telomere length. Due to the scarcity of existing data, the tertiary aim of this study is to replicate previous findings relating vagal tone to telomere length.

## 2 Materials and methods

### 2.1 Ethics statement

This study uses participant data from the sixth survey of the Tromsø Study (Tromsø6; Eggen et al., 2013) which was collected from 2007 to 2008. Seven surveys have been carried out so far. Tromsø6 was approved by the Data Inspectorate of Norway and the Regional Committee of Medical and Health Research Ethics, North Norway. Tromsø6 complies with the Declaration of Helsinki, International Ethical Guidelines for Biomedical Research Involving Human Subjects and the International Guidelines for Ethical Review of Epidemiological Studies. Participation was voluntary and each subject gave written informed consent prior to participation.

### 2.2 Participants

**FIGURE 2:**
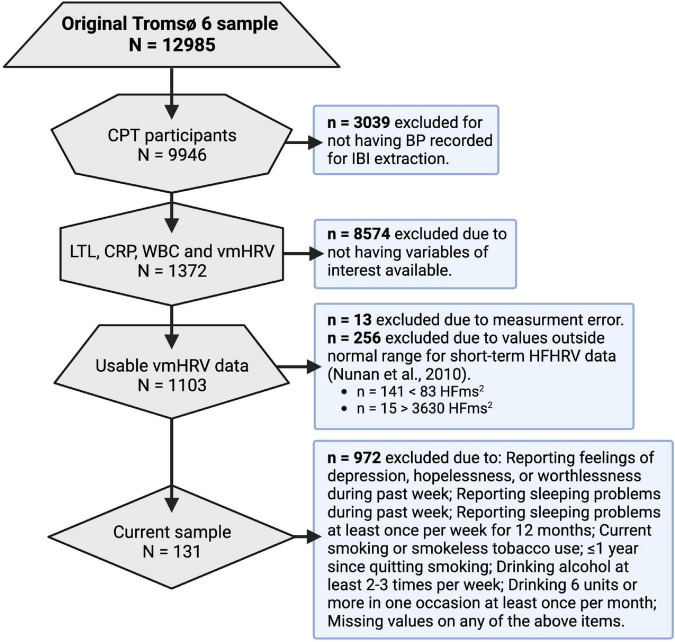
Data reduction process. CPT, cold-pressor test; BP, blood pressure; IBI, inter-beat interval; LTL, leukocyte telomere length; CRP, c reactive protein; WBC, white blood cell count; vmHRV, vagally mediated heart rate variability; HFms^2^, high frequency component HRV.

The Tromsø Study cohort consists of people living in the municipality of Tromsø, Norway, situated at 69° North (Jacobsen et al., 2012). Among the 70,000 people living in Tromsø when Tromsø6 was carried out, approximately 60,000 were living in the city-like town-center, while the remaining 10,000 were spread throughout the whole municipality (2,558 km^2^). Tromsø is a center of education, research, administration, and fishing related activities. It has a growing population that is predominantly Caucasians of Norwegian origin, but also includes a Sami minority. Tromsø may be considered as representative of a Northern European, white, urban population. A total of 12,984 (male = 6,054) participants aged 30–87 years enrolled in Tromsø6. Of these, 9,946 participated in a cold-pressor test (described in Schistad et al., 2017) where ultrashort recordings of blood pressure were conducted for inter-beat-interval (IBI) extraction and vmHRV quantification. A total of 1,372 participants that had vmHRV measures available also included measures of telomere length and CRP. Among those participants, only 1,103 had usable vmHRV data. 972 of these participants were excluded due to presence of confounders or missing data about confounders. The final sample consisted of 131 participants (mean age = 67.7, male = 81). See Figure 2 for an overview of the data reduction process, and the Section “2.6 Treatment of confounders” for more details. Characteristics of the 131 participants included in the final sample can be found in Table 1.

**TABLE 1:** Study sample characteristics (*N* = 131).

Characteristics	Number	Prevalence (%)	Mean	SD
Age			67.6	9.3
Sex (male)	81	61.8		
**Mental health**				
Depression	0	0		
**Sleeping problems**				
Sleeplessness 1–3 times per month during last 12 months	25	19.1		
Sleeping problem during last week, little complaint	23	17.6		
**Alcohol and tobacco use**				
Alcohol use 2–4 times a month	65	49.6		
Drinking 6 alcohol units or more less frequently than monthly	33	25.2		
Daily smoking	0	0		
Used to smoke daily	81	61.8		
Years since smoking cessation			23.0	14.3
Use snus/snuff/chewing tobacco	0	0		
Previous snus/snuff/chewing tobacco use	1	0.8		

### 2.3 Vagally mediated heart rate variability (vmHRV)

Resting blood pressure was measured non-invasively for 30 s using the Finometer Pro (Finometer, Finapres Medical System, Amsterdam, Netherlands) and HRV parameters were calculated as previously described (Bruehl et al., 2018). In short, IBIs were artifact corrected and time- and frequency domain HRV parameters were extracted using the RHRV package (Martínez et al., 2017). Artifacts were removed using a distribution-based algorithm (see Deegan et al., 2007a,b). The triangular interpolation of NN histogram and HRV index parameters was calculated using the shape of the histogram of the RR intervals and a bin width setting of 7.8125 ms. Fourier-based signal power calculation in the high frequency (0.15–0.4 Hz) bands (Task Force of the European Society of Cardiology and the North American Society of Pacing and Electrophysiology., 1996) were used to calculate high frequency-domain parameters.

Two commonly used indicators of prefrontally modulated vagal tone are the time domain measure root mean square of successive N-N differences (RMSSD) and the high-frequency (HF) component HRV measure (HFHRV; Task Force of the European Society of Cardiology and the North American Society of Pacing and Electrophysiology., 1996; Thayer et al., 2012). Using ultrashort recordings of IBIs to quantify HRV indices is promising in clinical settings if they can be applied to identify at-risk populations. We were mainly interested in HFHRV due to RMSSD possibly being influenced by sympathetic activation (Berntson et al., 2005) and vagal (Wang et al., 2003; Huston et al., 2006) and sympathetic (Madden et al., 1995; Vizi, 1998) inputs to the spleen having opposing effects on inflammatory marker expression. Furthermore, transcranial stimulation of the PFC leads to increases in HFHRV (Brunoni et al., 2013; Nikolin et al., 2017). Previous studies indicated that at least 20 s of recording are required for HFHRV variables to be reliable from ultrashort-term measurements (Shaffer and Ginsberg, 2017). Since our HRV indices were generated from 30 s recordings, HFHRV variables should be reliable. RMSSD and HFHRV are usually highly correlated (Goedhart et al., 2007). Due to applying ultrashort recordings for HRV calculation, RMSSD was included in the analysis to check for this correlation to assure that vmHRV indices were of good quality.

All measurements where the Standard Deviation of NN/(RR) intervals ≥ 150 were visually inspected (*n* = 107). 13 of the identified measurements were suspected of measurement error (e.g., due to displaying doubling and then halving of IBI length). These were excluded from the analysis. The normal range for HFHRV indices generated from ultrashort (20 s) recordings range from 18 to 3,630 (Nunan et al., 2010). 241 participants had HFHRV values below 18, while 15 participants had HFHRV values above 3,630. These participants were excluded from the analysis.

### 2.4 C reactive protein (CRP), leukocyte (WBC) count, and leukocyte telomere length (LTL) quantification

Blood sampling is described in Eggen et al. (2013). Examinations started at 9 a.m. in the morning. The participants were not required to fast prior to providing blood samples, but they were only allowed to drink water and black coffee during their visits. 50 ml blood samples were collected. Time since the last meal (hours) was recorded. Venipuncture was performed with subjects in a sitting position. A light tourniquet was used and released before sampling. After 30 min at room temperature, the coagulated samples were centrifuged at 2,000 *g* for 10 min, and the sera were transferred within 1 h to plastic tubes, and kept between 1 and 10°C. The blood samples were sent twice daily to the Department of Laboratory Medicine, University Hospital North Norway, Tromsø, which is an accredited laboratory (ISOstandard 17025).

C reactive protein was analyzed by a highly sensitive CRP method (particle-enhanced immunoturbidimetric assay). The analyses were performed on a Modal PPE auto-analyzer with reagents from Roche Diagnostics Norway AS.

For WBC, 5 ml of blood was collected into Vacutainer tubes containing EDTA as an anticoagulant (K3-EDTA 40 ll, 0.37 mol/L per tube), and analyzed within 12 h by an automated blood cell counter (Coulter CounterÒ, Coulter Electronics, Luton, UK and Coulter LH750, Nerliens Meszansky). Leukocyte telomere length (LTL) was analyzed according to established qPCR methods (O’Callaghan and Fenech, 2011) at McGill University in 2018 and the mean telomere length was quantified as arbitrary units.

### 2.5 Vagal neuroimmunomodulation index

To calculate the NIM, the z-transformed HFHRV variable was divided by z-transformed variables of the participants’ CRP to obtain the HFHRV/CRP ratio in accordance with previous research (Gidron et al., 2018; Jarczok et al., 2021).

### 2.6 Treatment of confounders

One major issue facing the literature on associations between vmHRV and inflammatory markers is the lack of controlling for confounding variables (Haensel et al., 2008; Papaioannou et al., 2013). Timing from last meal to blood samples being drawn (Marinac et al., 2015), disturbed sleep (Irwin et al., 2016), depression (Lee and Giuliani, 2019), and alcohol and tobacco use (e.g., Tibuakuu et al., 2017; van de Loo et al., 2020) are all associated with inflammatory marker expression as well as PFC activity and vmHRV. Information on sleeping problems, smoking habits, use of alcohol, and depression were obtained from self-administered screening questionnaires that were sent in the mail prior to the first visit (Eggen et al., 2013). Studies suggest that vmHRV is also dependent on sex (e.g., Koenig and Thayer, 2016) and age (Shaffer and Ginsberg, 2017), thus, sex and age were included as control variables in the analysis.

Items screening for depression included questions such as “Have you felt depressed or sad during the last week?” and “Have you felt hopelessness with regard to the future during the last week?”. Answers were scored on a 4-point scale ranging from 1 = no complaint, to 2 = little complaint, 3 = pretty much, and 4 = very much. Items measuring disturbed sleep included questions like “Have you had sleeping problems during the past week?” which was also used to screen for depression and measured on the same scale, and “How often have you suffered from sleeplessness during the last 12 months?” which were scored on a 4-point scale ranging from 1 = never, or just a few times a year, to 2 = one-three times per month, 3 = approximately once per week, and 4 = more than once a week.

Because the depression inventory was a screen and not a validated inventory measuring the presence and level of depression, individuals were excluded if they reported presence of the depressive moods and thoughts captured by the screen. In the present study, 972 participants were excluded due to: (1) reporting feelings of depression, hopelessness, or worthlessness during past week, (2) reporting having sleeping problems during past week, (3) reporting having sleeping problems at least once per week for 12 months, (4) reporting current smoking or smokeless tobacco use, (5) reporting it being ≤ 1 year since smoking cessation, (6) reporting drinking alcohol at least 2–3 times per week, (7) reporting drinking six units or more in one occasion at least once per month, and (8) missing values on any of the above items.

White blood cell count (WBC) has previously been found to be positively associated with LTL (Gutmajster et al., 2013; Neuner et al., 2015) and telomere length varies between different subtypes of leukocytes (Rufer et al., 1998; Wong et al., 2011). Considering positive associations between WBC and CRP (Sproston and Ashworth, 2018), and negative associations between CRP and LTL (Rode et al., 2014; Wong et al., 2014), WBC could serve as a bidirectional confounder in the relationship between CRP and LTL. It could also serve as a confounder in the relationship between vagal tone and LTL, as vagal and sympathetic activity differentially regulate leukocyte activity with vmHRV being negatively associated with WBC (Williams et al., 2019). WBC was therefore included as a control variable in the analysis.

### 2.7 Statistical analysis

First, the Shapiro–Wilk test of normality and confirmatory visual inspection of bar-graph distribution plots revealed that WBC, CRP, NIM index, and HRV variables were not normally distributed. Log10 transformation was performed on these variables prior to analysis. After log10 transformation, follow-up visual inspection and Shapiro–Wilk test confirmed normal distribution. All subsequent analyses were performed on the log10 transformed version of these variables.

Pearson correlations were conducted for HFHRV, RMSSD, the NIM index, CRP, WBC, LTL, and time since last meal to assess the relationship between the variables. Subsequent partial correlations were performed controlling for the influence of age, sex, WBC, and time since last meal. Results were presented in tables as Spearman correlation coefficients.

Separate linear regressions were performed using HFHRV, CRP, the NIM index, and WBC as predictors of LTL. Due to a positive correlation between WBC and LTL, and WBC and CRP in our sample, WBC was used as a control variable along with sex and age in subsequent linear hierarchical regressions by entering WBC, age, and sex as the first predictors in the model. Results for regressions were presented in tables. In Table 6, model 1 in the hierarchical regression assesses the effect of confounders on LTL. Model 2a assesses HFHRV as predictor of LTL along with confounders in model 1 Model 2b assesses CRP as predictor of LTL along with confounders in model 1. Model 2c simultaneously assesses HFHRV and CRP as predictors of LTL along with confounders in model 1.

**TABLE 2:** Descriptive statistics (*N* = 131).

Variables	Mean	SD	Min	Max
Mean RR	918.03	167.00	522.00	1254.00
HFHRV	452.21	663.59	85	3170.00
HFHRV_log	2.39	0.41	1.93	3.50
RMSSD	52.50	38.69	15.00	192.00
RMSSD_log	1.63	0.24	1.19	2.28
NIM	2.17	2.93	0.11	17.91
NIM_log	0.07	0.48	−0.93	1.25
CRP sensitive (mg/L)	2.44	2.28	0.00	13.00
CRP_log	0.23	0.36	−0.51	1.10
WBC (10 e9/l)	6.05	1.41	3.00	10.00
WBC_log	0.77	0.09	0.52	1.02
LTLarb	1.37	0.54	0.00	3.00
Time since last meal (hours)	3.33	1.88	0.00	9.00

RR, R-to-R peak interval in milliseconds; HR, heart rate; HFHRV, high frequency component heart rate variability; _log, log10-transformed variable; RMSSD, root mean square of successive N-N differences; NIM, neuroimmunomodulation index; CRP, c reactive protein; WBC, white blood cell count; LTLarb, leukocyte telomere length arbitrary units.

**TABLE 3:** Pearson correlations (*N* = 131).

Variables	1	2	3	4	5	6	7
(1) HFHRV_log	−						
(2) RMSSD_log	0.821***	−					
(3) NIM_log	0.515***	0.439***	−				
(4) CRP_log	0.047	0.023	-0.825***	−			
(5) WBC_log	-0.029	-0.114	-0.213*	0.232**	−		
(6) LTLarb	0.284***	0.186*	0.228**	-0.077	0.225**	−	
(7) Time since last meal	0.032	0.093	0.106	-0.096	-0.058	0.097	−

All correlations are 2-tailed. HFHRV, high frequency component heart rate variability; _log, log10-transformed variable; RMSSD, root mean square of successive N-N differences; NIM, vagal neuroimmunomodulation index; CRP, c reactive protein; WBC, white blood cell count; LTLarb, leukocyte telomere length arbitrary units.

*p < 0.050; **p < 0.010; ***p < 0.001.

**TABLE 4:** Partial correlations (*N* = 131).

Variables	1	2	3	4
(1) HFHRV_log	−			
(2) NIM_log	0.535***	−		
(3) CRP_log	0.010	-0.833***	−	
(4) LTLarb	0.330***	0.300***	-0.135	−

All correlations are controlling for age, sex, WBC, and time since last meal. All correlations are 2-tailed. HFHRV, high frequency component heart rate variability; _log, log10-transformed variable; NIM, vagal neuroimmunomodulation index; CRP, c reactive protein; WBC, white blood cell count; LTL, leukocyte telomere length arbitrary units.

***p < 0.001.

**TABLE 5:** Linear regression for HFHRV, CRP, NIM index, WBC, and LTL (*N* = 131).

Predictor	Dependent variable	β	*p*	*R^2^_*adj*_*	*F*(1)
HFHRV_log	LTLarb	0.284	0.001	0.073	11.30
HFHRV_log	CRP	0.047	0.595	-0.006	0.28
CRP_log	LTLarb	-0.077	0.383	-0.002	0.76
NIM_log	LTLarb	0.228	0.009	0.045	7.09
WBC_log	LTLarb	0.225	0.010	0.043	6.89

HFHRV_log, high frequency component heart rate variability; CRP_log, c reactive protein; NIM, vagal neuroimmunomodulation index; WBC_log, white blood cell count; _log, log10-transformed variable; LTL, leukocyte telomere length arbitrary units.

**TABLE 6:** Hierarchical regression models for HFHRV and CRP as predictors of LTL (*N* = 131).

Model	β	*p*	*R^2^_*adj*_*	*F*(df)
WBC_log	0.227	0.008	0.075	*F*(3) = 4.51
Age	-0.143	0.093		
Sex	-0.160	0.061		
**Model 2a**				
WBC_log	0.236	0.004	0.177	*F*(4) = 7.98
Age	-0.201	0.014		
Sex	-0.170	0.035		
HFHRV_log	0.331	<0.001		
**Model 2b**				
WBC_log	0.258	0.003	0.084	*F*(4) = 3.97
Age	-0.125	0.144		
Sex	-0.169	0.047		
CRP_log	-0.131	0.137		
**Model 2c**				
WBC_log	0.270	0.001	0.190	*F*(5) = 7.08
Age	-0.182	0.027		
Sex	-0.180	0.025		
HFHRV_log	0.335	<0.001		
CRP_log	-0.142	0.086		

HFHRV_log, high frequency component heart rate variability; CRP_log, c reactive protein; WBC_log, white blood cell count; _log, log10-transformed variable; LTL, leukocyte telomere length arbitrary units.

To determine whether HFHRV or the NIM index were better indicators of telomere length in the present sample, a follow-up analysis of covariance (ANCOVA) analysis was conducted with high and low NIM index (median split) and high and low HFHRV (median split) simultaneously entered as fixed factors, WBC as covariate, and LTL as dependent variable. Dividing NIM index (Gidron et al., 2018; Jarczok et al., 2021) and HFHRV in groups (Hansen et al., 2003, 2009; Pu et al., 2010; Williams et al., 2017) with high and low values is a common approach. Effect sizes were calculated as partial eta squared (η^2^_*p*_). Pairwise comparisons among estimated marginal means were used to assess significant differences between groups and was reported as estimated marginal means-based mean difference (MD_*EM*_) and Bonferroni adjusted *p*-values (*p*_*bonf*_).

Data was analyzed using SPSS version 25 (IBM Corp, 2017).

## 3 Results

### 3.1 Participant characteristics and descriptive statistics of vmHRV, NIM index, and biomarkers

The demographic characteristics of the studied population (*N* = 131) are shown in Table 1. Table 2 provides descriptive statistics for extracted vmHRV indices, the NIM index, LTL, CRP, and WBC before and after log10 transformation, and time since last meal (hours).

### 3.2 Relationship between vmHRV, CRP, WBC, and LTL

Table 3 provides an overview of the Pearson’s correlation results. RMSSD and HFHRV were highly correlated (*p* < 0.001). HFHRV was significantly associated with LTL (*p* < 0.001) but not CRP (*p* = 0.595). HFHRV was not significantly associated with WBC (*p* = 0.742). CRP was not significantly associated with LTL (*p* = 0.383). CRP was significantly associated with WBC (*p* = 0.008). WBC was significantly associated with LTL (*p* = 0.010). Time since last meal was not associated with any variables.

Table 4 provides the results for the partial correlations controlling for sex, age, WBC, and time since last meal. When controlling for the influence of sex, age, WBC, and time since last meal, HFHRV was significantly associated with the NIM index (*p* < 0.001), and LTL (*p* < 0.001), but not CRP (*p* = 0.914). CRP was not associated with LTL (*p* = 0.181).

Tables 5, 6 shows the initial regression analyses for HFHRV and CRP as predictors of LTL, and the hierarchical multiple regression analyses when controlling for age, sex, and WBC, respectively. In the first linear regression analysis, HFHRV was a significant predictor of LTL (*p* = 0.001). In the hierarchical multiple regression controlling for age, sex, and WBC (model 2a), HFHRV was a significant predictor of LTL (*p* < 0.001).

As indicated in the initial correlation analyses, HFHRV was not a significant predictor of CRP alone (*p* = 0.595), nor when controlling for age, sex, and WBC [β = 0.032, *p* = 0.709, *R^2^_*adj*_* = 0.050, *F*(4) = 2.722], and CRP was not a significant predictor of LTL in the initial analyses (*p* = 0.383), when controlling for age, sex, and WBC (*p* = 0.137), or when including HFHRV in the model (*p* = 0.086).

### 3.3 Relationship between the NIM index and LTL

In the initial Spearman correlation analysis, the NIM index was significantly associated with LTL (*p* = 0.009), CRP (*p* < 0.001), and WBC (*p* = 0.015). In the partial correlation analysis controlling for age, sex, WBC, and time since last meal, the NIM index was significantly associated with LTL (*p* < 0.001).

**FIGURE 3:**
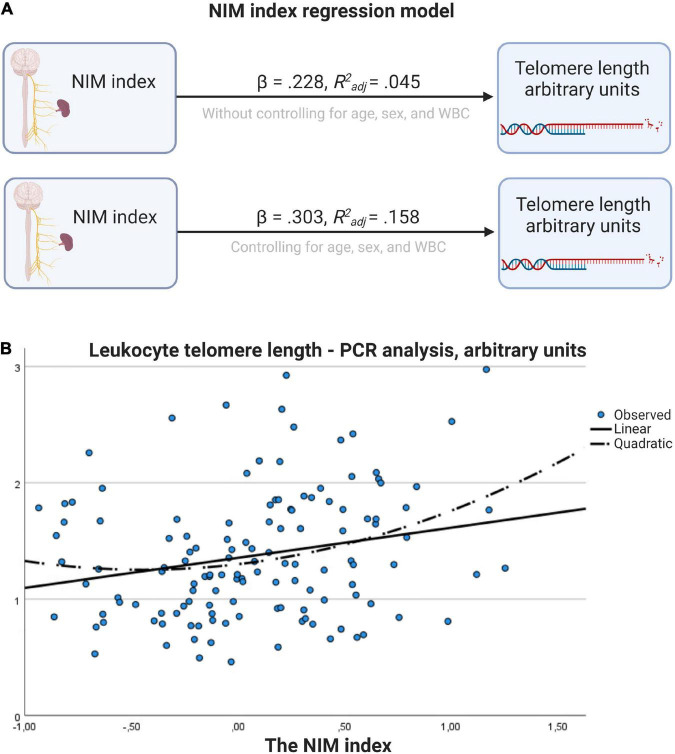
Regression model for the relationship between NIM index and telomere length. **(A)** Results with and without controlling for age, sex, and WBC. **(B)** Scatter plot with linear and quadratic regression line. Figure created with BioRender.com.

Tables 5, 7 shows the initial regression analyses for the NIM index as predictor of LTL, and the hierarchical multiple regression analyses controlling for confounders, respectively. In the first linear regression analysis, the NIM index was a significant predictor of LTL (*p* = 0.009). In the hierarchical multiple regression controlling for WBC, the NIM index was a significant predictor of LTL (*p* < 0.001). Figures 3A, B shows the regression model for the relationship between the NIM index and LTL.

**TABLE 7:** Hierarchical regression models for the NIM index as predictor of LTL (*N* = 131).

Model	β	*p*	*R^2^_*adj*_*	*F*(df)
**Model 1**				
WBC_log	0.227	0.008	0.075	*F*(3) = 4.51
Age	-0.143	0.093		
Sex	-0.160	0.061		
**Model 2**				
WBC_log	0.292	<0.001	0.158	*F*(4) = 7.07
Age	-0.139	0.086		
Sex	-0.184	0.024		
NIM_log	0.303	<0.001		

WBC_log, white blood cell count; NIM, vagal neuroimmunomodulation index; _log, log10-transformed variable; LTL, leukocyte telomere length arbitrary units.

After confirming that WBC was not significantly different between high and low HFHRV or high and low NIM index groups, a follow-up ANCOVA analysis was conducted with high and low NIM index groups and high and low HFHRV groups as fixed factors, WBC as covariate, and LTL as dependent variable. In the test of between-subjects effects, WBC had a significant effect on LTL [*F*(1, 126) = 10.79, *p* = 0.001, η^2^_*p*_ = 0.079]. LTL was significantly different between NIM index groups [*F*(1, 126) = 9.01, *p* = 0.003, η^2^_*p*_ = 0.067]. LTL was not significantly different between HFHRV groups [*F*(1, 126) = 2.63, *p* = 0.107, η^2^_*p*_ = 0.020]. The interaction between NIM index groups and HFHRV groups did not have a significant effect on LTL [*F*(1, 126) = 0.17, *p* = 0.687, η^2^_*p*_ = 0.001].

**FIGURE 4:**
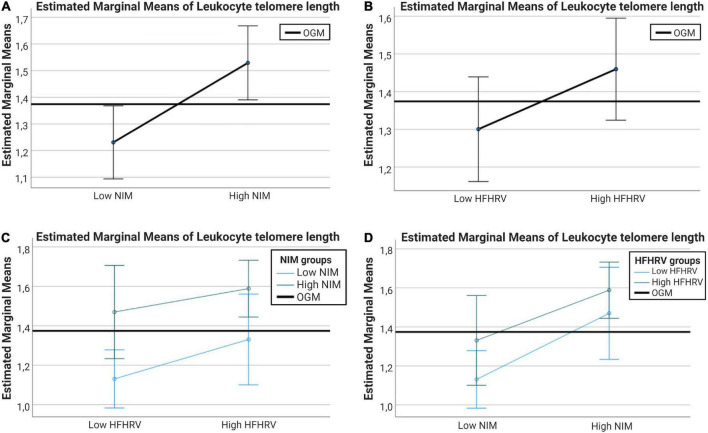
Interval plots showing the differences in telomere length between NIM index groups and between HFHRV groups. **(A)** Interval plots for differences in LTL between high and low NIM index groups. **(B)** Interval plots for differences in LTL between high and low HFHRV groups. **(C)** Interval plots plotting differences in LTL between high and low HFHRV groups according to NIM index group. **(D)** Interval plots plotting differences in LTL between high and low NIM index groups according to HFHRV groups. Error bars are 95% confidence intervals. Covariates appearing in the models are evaluated at the following values: WBC = 0.7704. OGM, observed grand mean; WBC, white blood cell count; NIM, vagal neuroimmunomodulation index; HFHRV, high frequency component heart rate variability.

Pairwise comparisons showed that individuals with low NIM index values had significantly shorter LTL compared to individuals with high NIM index values (MD_*EM*_ = −0.298, *p*_*bonf*_ = 0.003). Figures 4A–D shows interval plots for covariate-adjusted differences in LTL between HFHRV groups and NIM index groups. Confidence intervals (indicated by the whiskers in the interval plots in Figure 4) are smaller for the NIM index groups [Figure 4A than for HFHRV groups (Figure 4B)]. Plotting differences in LTL between high and low HFHRV according to NIM index groups indicate that individuals with high NIM index have longer LTL regardless of being in the low HFHRV group (Figure 4C), but individuals with high HFHRV have longer telomeres only if they are in the high NIM group (Figure 4D).

## 4 Discussion

This is the first study to assess inflammation as a mediator in the relationship between vmHRV and telomere length, and to assess the relationship between the NIM index, a recently proposed index for vagal neuroimmunomodulation (Gidron et al., 2018), a possible indicator of PAS axis activity, and telomere length. It is also the first paper to conceptualize the neural substrate facilitating the relationships between the prefrontal component of the central autonomic network and its influence on vagally modulated splenogenic inflammation as the PAS axis.

In our initial correlation analysis, we found that vmHRV was positively associated with telomere length, while CRP was not. White blood cell count was previously found to be positively associated with telomere length (Gutmajster et al., 2013; Neuner et al., 2015) and CRP (Sproston and Ashworth, 2018). Because these relationships were found in our sample as well, we conducted partial correlations controlling for this possible bidirectional confounder along with sex, age, and time since last meal. VmHRV remained positively associated with telomere length, but relationships between vmHRV and CRP, and CRP and telomere length remained insignificant. This was true also in our regression analyses. Thus, in the present sample, vmHRV was not associated with CRP, which is in contrast to the findings of Williams et al. (2019). Moreover, while the association between vmHRV and telomere length is in line with previous findings (Zalli et al., 2014; Perseguini et al., 2015; Streltsova et al., 2017; Woody et al., 2017; Wilson et al., 2019; Shahane et al., 2020), CRP did not mediate the relationship between vmHRV and telomere length, in contrast to the postulated hypothesis (Ask et al., 2018).

A possible explanation for the discrepancy between our findings and the findings of Williams et al. (2019) could perhaps be attributed to our strict criteria for inclusion to eliminate known confounders in the relationship between vagal tone and inflammation (Haensel et al., 2008; Papaioannou et al., 2013). Of note, in Williams et al. (2019), it was IL-6 that showed the strongest association with vmHRV, while CRP showed the second strongest association to vmHRV. The role of CRP in inflammation is not straightforward with sometimes opposing effects being ascribed to pentameric native CRP (nCRP) versus monomeric CRP (mCRP), the former isoform having primarily anti-inflammatory effects while mCRP having mainly pro-inflammatory effects (Sproston and Ashworth, 2018). In our study, CRP isoform was not specified, thus, the mediation analysis might therefore not provide sufficient sensitivity to properly examine the relationships of interest. Transcriptional induction of CRP is downstream of IL-6 (Boras et al., 2014). It is thus hard to say how the results would have been if CRP type had been specified or if IL-6 had been assessed instead. It is worth mentioning that in the preliminary analyses reported in the preprint for the present study (Ask and Sütterlin, 2022, preprint), an association was found between vmHRV and CRP when including participants with vmHRV outside the normal range.

One thing to keep in mind when using HRV indicators of vagal tone is that, while the sympathetic nervous system acts as a unit, the parasympathetic nervous system does not. Thus, there is arguably going to be some level of dissociation between vagal input to the heart and vagal input to the spleen, including reflex circuit activity originating from central nervous system inputs other than the PFC. The NIM index, which is calculated by the ratio of vmHRV to CRP has been suggested as a biomarker for vagal neuroimmunomodulation (Gidron et al., 2018). Previous studies found that the NIM index is associated with cancer survival and all-cause mortality (Gidron et al., 2018; Jarczok et al., 2021). In our study, the NIM index was positively associated with telomere length both with and without controlling for age, sex, and WBC. Interestingly, when controlling for confounders, both sex and age became significant predictors of telomere length only after adding vmHRV to the model. When assessing the NIM index as a predictor of telomere length, sex (but not age) became a significant predictor of telomere length after adding NIM index to the model. This latter finding is in line with the concept of “inflammaging” which posits that inflammation is a driver of biological aging (Ferrucci and Fabbri, 2018), and may further suggest that the NIM index captures age-related inflammatory processes not captured when assessing vmHRV in isolation.

In a follow-up analysis, after dividing participants in groups according to having high and low vmHRV (Hansen et al., 2003, 2009; Pu et al., 2010; Williams et al., 2017), and having high and low NIM index values (Gidron et al., 2018; Jarczok et al., 2021), we found that telomere length was significantly different between individuals with high and low NIM index values, but not individuals with high and low vmHRV. Interval plots indicated that telomere length was longer if vagal neuroimmunomodulation was high regardless of vmHRV being high or low. This is also in line with the notions forwarded by Gidron et al. (2018), which were that inflammation by itself is not a sufficient indicator of vagal neuroimmunomodulation, and vagal activity by itself is not a sufficient indicator of inflammation levels. The positive association between the NIM index and telomere length is in support of the NISIM (Ask et al., 2018) and suggests that the NIM index is a better indicator of PAS axis activity than vmHRV and inflammation considered in isolation. Although the high frequency component of vagal tone used to quantify the NIM index in the present study is associated with PFC activity (Brunoni et al., 2013; Nikolin et al., 2017), more studies are needed to determine the degree to which NIM index values reflect PFC activity versus reflex circuits of other neuronal origins. This is especially important for indices quantified from ultrashort term recordings as they are easy to implement in clinical practice.

Previous studies suggested that the vagus nerve should be considered when understanding age-related pathologies (De Couck et al., 2012). The NISIM argues that PAS axis dysregulation will accelerate aging and the transition from health to age-related pathology (Ask et al., 2018). The role of amyloid plaque in Alzheimer’s disease is undecided with accumulating evidence indicating that it might not be the pathogenic trigger driving the neurodegenerative cascade (e.g., Sturchio et al., 2021). Alternative explanatory mechanisms that trigger and interact with known antecedents to neurodegenerative pathology are thus needed. Studies on senescent cells in the brain may offer plausible causative factors for neurodegeneration (Baker et al., 2011; Chinta et al., 2015). Recent studies indicated that increases in peripheral inflammation is a prodromal indicator of dementia, with greater cognitive impairment being associated with increased levels of IL-6 and TNF-α in Alzheimer’s and Parkinson’s disease [King et al., 2018; Wood, 2018; see Maletic and Raison (2014) for a review of how peripheral inflammatory signals can be transported into the brain in psychiatric disease]. While the NIM index is associated with survival in cancer patients and all-cause mortality (Gidron et al., 2018; Jarczok et al., 2021), no studies have looked at its role as predictor of age-related pathology such as Alzheimer’s disease. Telomere attrition is an antecedent to cellular senescence (Campisi, 2000, 2013; Brand, 2019; Coluzzi et al., 2019) and shorter telomeres has been identified as a risk factor for developing Alzheimer’s disease (Hackenhaar et al., 2021). While our findings indicate that the NIM index is associated with telomere length, future studies should determine whether the NIM index can serve as a predictor of cognitive decline. This will help determine its potential use case as a biomarker for identifying vulnerable populations at risk for age related cognitive decline.

### 4.1 Limitations and future perspectives

C reactive protein subtype was not specified in the present study which may have impacted results. Thus, the study should be repeated with CRP subtype specified. Future studies should include other inflammatory markers, particularly IL-6 as this marker may provide a better proxy for the inflammatory effects of a dysregulated PAS axis (Williams et al., 2019) and may also be a better inflammatory marker for NIM index calculation (Jarczok et al., 2021). Moreover, while participant examinations started at 9 a.m., participants were allowed to come in later (Eggen et al., 2013), thus, it is reasonable to suggest that CRP levels could be influenced by variations in when blood samples were drawn. The present study used ultrashort recordings to calculate vmHRV. While ultrashort recordings using the Finometer Pro serves as a promising approach in clinical settings it is not without its downsides and the finding in the present study should be interpreted with caution. At least 20 s of recording is required for HFHRV measures to be reliable (Nunan et al., 2010). The fact that we used 30 s of recordings and that RMSSD and HFHRV indices were highly correlated indicate that the measures were of good quality (Goedhart et al., 2007). Future studies should use 5-min recordings to quantify vmHRV to reduce effects owing to suboptimal IBI measurements. Telomere length, CRP, and IBIs were not measured on the same day which may have reduced effect sizes. A final limitation owes to the correlational nature of the study as it does not provide direct evidence for the NISIM. Evidence suggests that telomere dysfunction and senescence can occur irrespective of telomere length (Victorelli and Passos, 2017). Longitudinal studies are needed to assess the directional relationship between prefrontally modulated vagal tone, inflammatory markers, telomere length, and transition from health to age-related pathology.

## 5 Conclusion

This study is an important first step toward understanding the anatomical-to-molecular mechanisms that relate individual differences in neurocognitive stress regulation capacity to PAS axis-related induction of cellular senescence and age-related pathology. Our results suggest that PAS axis activity is associated with telomere length, which may indicate that PAS axis dysregulation leads to increased levels of inflammation and subsequently ROS induced telomere damage. Based on the findings of our study, we argue that the NIM index is a sensitive proxy for PAS axis activity and could serve as a useful indicator when assessing PAS axis-related effects on antecedents to cellular senescence. Future studies should assess the relationship between indicators of prefrontally modulated vagal tone, IL-6, CRP subtype, and telomere length as well as more direct markers of prefrontal activity, oxidative telomere damage, and telomere damage-induced cellular senescence.

## Data availability statement

The dataset analyzed in the current study is not publicly available, but access can be requested from the Tromsø Study (https://uit.no/research/tromsostudy/project?pid=709148). To respect terms of use and protect human privacy, the authors cannot directly share the dataset. Requests to access these datasets should be directed to Tromsø Study, tromsous@uit.no.

## Ethics statement

The studies involving human participants were reviewed and approved by the Data Inspectorate of Norway and the Regional Committee of Medical and Health Research Ethics, North Norway. The patients/participants provided their written informed consent to participate in this study.

## Author contributions

TA: writing of the original draft, data reduction, analysis, review, and editing. SS: writing, review, and editing. Both authors approved the final version of the manuscript.

## References

[B1] Ask T. F. Lugo R. G. Sütterlin S. (2018). The neuro-immuno-senescence integrative model (NISIM) on the negative association between parasympathetic activity and cellular senescence. *Front. Neurosci.* 12:726. 10.3389/fnins.2018.00726 30369866 PMC6194361

[B2] Ask T. F. Sütterlin S. (2022). Prefrontally modulated vagal tone inhibits inflammatory responses to prevent telomere damage in healthy participants. *bioRxiv* [Preprint]. 10.1101/2022.02.17.480574

[B3] Baker D. J. Wijshake T. Tchkonia T. LeBrasseur N. K. Childs B. G. van de Sluis B. (2011). Clearance of p16Ink4a-positive senescent cells delays ageing-associated disorders. *Nature* 479 232–236. 10.1038/nature10600 22048312 PMC3468323

[B4] Berntson G. G. Lozano D. L. Chen Y. J. (2005). Filter properties of root mean square successive difference (RMSSD) for heart rate. *Psychophysiology* 42 246–252. 10.1111/j.1469-8986.2005.00277.x 15787862

[B5] Bissell M. J. Kenny P. A. Radisky D. C. (2005). Microenvironmental regulators of tissue structure and function also regulate tumor induction and progression: The role of extracellular matrix and its degrading enzymes. *Cold Spring Harb. Symp. Quant. Biol.* 70 343–356. 10.1101/sqb.2005.70.013 16869771 PMC3004779

[B6] Boras E. Slevin M. Alexander M. Y. Aljohi A. Gilmore W. Ashworth J. (2014). Monomeric C-reactive protein and Notch-3 co-operatively increase angiogenesis through PI3K signalling pathway. *Cytokine* 69 165–179. 10.1016/j.cyto.2014.05.027 24972386

[B7] Brand T. (2019). Length doesn’t matter—telomere damage triggers cellular senescence in the ageing heart. *EMBO J.* 38:e101571. 10.15252/embj.2019101571 30770342 PMC6396151

[B8] Bruehl S. Olsen R. B. Tronstad C. Sevre K. Burns J. W. Schirmer H. (2018). Chronic pain-related changes in cardiovascular regulation and impact on comorbid hypertension in a general population: The Tromsø study. *Pain* 159 119–127. 10.1097/j.pain.0000000000001070 28953193

[B9] Brunoni A. R. Vanderhasselt M. A. Boggio P. S. Fregni F. Dantas E. M. Mill J. G. (2013). Polarity- and valence-dependent effects of prefrontal transcranial direct current stimulation on heart rate variability and salivary cortisol. *Psychoneuroendocrinology* 38 58–66. 10.1016/j.psyneuen.2012.04.020 22626867

[B10] Campisi J. (2000). Cancer, aging and cellular senescence. *In Vivo* 14 183–188. 10757076

[B11] Campisi J. (2013). Aging, cellular senescence, and cancer. *Ann. Rev. Physiol.* 75 685–705. 10.1146/annurev-physiol-030212-183653 23140366 PMC4166529

[B12] Chand T. Li M. Jamalabadi H. Wagner G. Lord A. Alizadeh S. (2020). Heart rate variability as an index of differential brain dynamics at rest and after acute stress induction. *Front. Neurosci.* 14:645. 10.3389/fnins.2020.00645 32714132 PMC7344021

[B13] Chiang J. K. Kuo T. B. Fu C. H. Koo M. (2013). Predicting 7-day survival using heart rate variability in hospice patients with non-lung cancers. *PLoS One* 8:e69482. 10.1371/journal.pone.0069482 23936027 PMC3720672

[B14] Chinta S. J. Woods G. Rane A. Demaria M. Campisi J. Andersen J. K. (2015). Cellular senescence and the aging brain. *Exp. Gerontol.* 68 3–7. 10.1016/j.exger.2014.09.018 25281806 PMC4382436

[B15] Coluzzi E. Leone S. Sgura A. (2019). Oxidative stress induces telomere dysfunction and senescence by replication fork arrest. *Cells* 8:19. 10.3390/cells8010019 30609792 PMC6356380

[B16] De Couck M. Mravec B. Gidron Y. (2012). You may need the vagus nerve to understand pathophysiology and to treat diseases. *Clin. Sci.* (London, England: 1979), 122, 323–328. 10.1042/CS20110299 22150254

[B17] De Couck M. van Brummelen D. Schallier D. De Grève J. Gidron Y. (2013). The relationship between vagal nerve activity and clinical outcomes in prostate and non-small cell lung cancer patients. *Oncol. Rep.* 30 2435–2441. 10.3892/or.2013.2725 24026706

[B18] Deegan B. M. O’Connor M. Lyons D. O’Laighin G. (2007a). A new blood pressure and heart rate signal analysis technique to assess Orthostatic Hypotension and its subtypes. *Ann. Int. Conf. IEEE Eng. Med. Biol. Soc.* 2007 935–938. 10.1109/IEMBS.2007.4352445 18002111

[B19] Deegan B. M. O’Connor M. Lyons D. O’Laighin G. (2007b). Development and evaluation of new blood pressure and heart rate signal analysis techniques to assess orthostatic hypotension and its subtypes. *Physiol. Meas.* 28:N87–N102. 10.1088/0967-3334/28/11/N01 17978417

[B20] Eggen A. E. Mathiesen E. B. Wilsgaard T. Jacobsen B. K. Njølstad I. (2013). The sixth survey of the Tromsø study (Tromsø 6) in 2007–08: Collaborative research in the interface between clinical medicine and epidemiology: Study objectives, design, data collection procedures, and attendance in a multipurpose population-based health survey. *Scand. J. Public Health* 41 65–80. 10.1177/1403494812469851 23341355

[B21] Epel E. Daubenmier J. Moskowitz J. T. Folkman S. Blackburn E. (2009). Can meditation slow rate of cellular aging? cognitive stress, mindfulness, and telomeres. *Ann. N. Y. Acad. Sci.* 1172 34–53. 10.1111/j.1749-6632.2009.04414.x 19735238 PMC3057175

[B22] Epel E. S. Blackburn E. H. Lin J. Dhabhar F. S. Adler N. E. Morrow J. D. (2004). Accelerated telomere shortening in response to life stress. *Proc. Natl. Acad. Sci. U.S.A.* 101 17312–17315. 10.1073/pnas.0407162101 15574496 PMC534658

[B23] Ferrucci L. Fabbri E. (2018). Inflammageing: Chronic inflammation in ageing, cardiovascular disease, and frailty. *Nat. Rev. Cardiol.* 15 505–522. 10.1038/s41569-018-0064-2 30065258 PMC6146930

[B24] Fitzpatrick A. L. Kronmal R. A. Gardner J. P. Psaty B. M. Jenny N. S. Tracy R. P. (2007). Leukocyte telomere length and cardiovascular disease in the cardiovascular health study. *Am. J. Epidemiol.* 165 14–21. 10.1093/aje/kwj346 17043079

[B25] Gidron Y. De Couck M. Schallier D. De Greve J. Van Laethem J. L. Maréchal R. (2018). The relationship between a new biomarker of vagal neuroimmunomodulation and survival in two fatal cancers. *J. Immunol. Res.* 2018:4874193. 10.1155/2018/4874193 29854838 PMC5964597

[B26] Goedhart A. D. van der Sluis S. Houtveen J. H. Willemsen G. de Geus E. J. (2007). Comparison of time and frequency domain measures of RSA in ambulatory recordings. *Psychophysiology* 44 203–215. 10.1111/j.1469-8986.2006.00490.x 17343704

[B27] Gutmajster E. Witecka J. Wyskida M. Koscinska-Marczewska J. Szwed M. Owczarz M. (2013). Telomere length in elderly caucasians weakly correlates with blood cell counts. *Sci. World J.* 2013:153608. 10.1155/2013/153608 24453794 PMC3881685

[B28] Hackenhaar F. S. Josefsson M. Adolfsson A. N. Landfors M. Kauppi K. Hultdin M. (2021). Short leukocyte telomeres predict 25-year Alzheimer’s disease incidence in non-APOE ε4-carriers. *Alzheimer’s Res. Ther.* 13:130. 10.1186/s13195-021-00871-y 34266503 PMC8283833

[B29] Haensel A. Mills P. J. Nelesen R. A. Ziegler M. G. Dimsdale J. E. (2008). The relationship between heart rate variability and inflammatory markers in cardiovascular diseases. *Psychoneuroendocrinology* 33 1305–1312. 10.1016/j.psyneuen.2008.08.007 18819754 PMC4266571

[B30] Hansen A. L. Johnsen B. H. Thayer J. F. (2003). Vagal influence on working memory and attention. *Int. J. Psychophysiol.* 48 263–274. 10.1016/s0167-8760(03)00073-4 12798986

[B31] Hansen A. L. Johnsen B. H. Thayer J. F. (2009). Relationship between heart rate variability and cognitive function during threat of shock. *Anxiety Stress Coping* 22 77–89. 10.1080/10615800802272251 18781457

[B32] Hildebrandt L. K. McCall C. Engen H. G. Singer T. (2016). Cognitive flexibility, heart rate variability, and resilience predict fine-grained regulation of arousal during prolonged threat. *Psychophysiology* 53 880–890. 10.1111/psyp.12632 26899260

[B33] Huston J. M. Ochani M. Rosas-Ballina M. Liao H. Ochani K. Pavlov V. A. (2006). Splenectomy inactivates the cholinergic antiinflammatory pathway during lethal endotoxemia and polymicrobial sepsis. *J. Exp. Med.* 203 1623–1628. 10.1084/jem.20052362 16785311 PMC2118357

[B34] IBM Corp (2017). *Released 2017. IBM SPSS Statistics for Windows, Version 25.0*. (Armonk, NY: IBM Corp).

[B35] Irwin M. R. Olmstead R. Carroll J. E. (2016). Sleep disturbance, sleep duration, and inflammation: A systematic review and meta-analysis of cohort studies and experimental sleep deprivation. *Biol. Psychiatry* 80 40–52. 10.1016/j.biopsych.2015.05.014 26140821 PMC4666828

[B36] Jacobsen B. K. Eggen A. E. Mathiesen E. B. Wilsgaard T. Njølstad I. (2012). Cohort profile: The Tromsø study. *Int. J. Epidemiol.* 41 961–967. 10.1093/ije/dyr049 21422063 PMC3429870

[B37] Jarczok M. N. Koenig J. Thayer J. F. (2021). Lower values of a novel index of Vagal-Neuroimmunomodulation are associated to higher all-cause mortality in two large general population samples with 18 year follow up. *Sci. Rep.* 11:2554. 10.1038/s41598-021-82168-6 33510335 PMC7844270

[B38] Kane A. E. Sinclair D. A. (2019). Epigenetic changes during aging and their reprogramming potential. *Crit. Rev. Biochem. Mol. Biol.* 54 61–83. 10.1080/10409238.2019.1570075 30822165 PMC6424622

[B39] King E. O’Brien J. T. Donaghy P. Morris C. Barnett N. Olsen K. (2018). Peripheral inflammation in prodromal Alzheimer’s and lewy body dementias. *J. Neurol. Neurosurg. Psychiatry* 89 339–345. 10.1136/jnnp-2017-317134 29248892 PMC5869446

[B40] Koenig J. Thayer J. F. (2016). Sex differences in healthy human heart rate variability: A meta-analysis. *Neurosci. Biobehav. Rev.* 64 288–310. 10.1016/j.neubiorev.2016.03.007 26964804

[B41] Kroenke C. H. Epel E. Adler N. Bush N. R. Obradovic J. Lin J. (2011). Autonomic and adrenocortical reactivity and buccal cell telomere length in kindergarten children. *Psychosom. Med.* 73 533–540. 10.1097/PSY.0b013e318229acfc 21873585 PMC3212037

[B42] Lee C. H. Giuliani F. (2019). The role of inflammation in depression and fatigue. *Front. Immunol.* 10:1696. 10.3389/fimmu.2019.01696 31379879 PMC6658985

[B43] Madden K. S. Sanders V. M. Felten D. L. (1995). Catecholamine influences and sympathetic neural modulation of immune responsiveness. *Annu. Rev. Pharmacol. Toxicol.* 35 417–448. 10.1146/annurev.pa.35.040195.002221 7598501

[B44] Maletic V. Raison C. (2014). Integrated neurobiology of bipolar disorder. *Front. Psychiatry* 5:98. 10.3389/fpsyt.2014.00098 25202283 PMC4142322

[B45] Marinac C. R. Sears D. D. Natarajan L. Gallo L. C. Breen C. I. Patterson R. E. (2015). Frequency and circadian timing of eating may influence biomarkers of inflammation and insulin resistance associated with breast cancer risk. *PLoS One* 10:e0136240. 10.1371/journal.pone.0136240 26305095 PMC4549297

[B46] Martínez C. A. G. Quintana A. O. Vila X. A. Touriño M. J. L. Rodríguez-Liñares L. Presedo J. M. R. (2017). *Heart rate variability analysis with the R package RHRV. Use R!.* Berlin: Springer.

[B47] Masi S. Nightingale C. M. Day I. N. Guthrie P. Rumley A. Lowe G. D. (2012). Inflammation and not cardiovascular risk factors is associated with short leukocyte telomere length in 13- to 16-year-old adolescents. *Arterioscler. Thromb. Vasc. Biol.* 32 2029–2034. 10.1161/ATVBAHA.112.250589 22679311

[B48] Morey J. N. Boggero I. A. Scott A. B. Segerstrom S. C. (2015). Current directions in stress and human immune function. *Curr. Opin. Psychol.* 5 13–17. 10.1016/j.copsyc.2015.03.007 26086030 PMC4465119

[B49] Mouton C. Ronson A. Razavi D. Delhaye F. Kupper N. Paesmans M. (2012). The relationship between heart rate variability and time-course of carcinoembryonic antigen in colorectal cancer. *Auton. Neurosci.* 166 96–99. 10.1016/j.autneu.2011.10.002 22070982

[B50] Mouton C. Ronson A. Razavi D. Nougaret J. Delhaye F. Hendliez A. (2010). The relationship between heart rate variability and prognosis in two cancers. *Int. J. Mol. Med.* 26:S9–S9.

[B51] Neuner B. Lenfers A. Kelsch R. Jäger K. Brüggmann N. van der Harst P. (2015). Telomere length is not related to established cardiovascular risk factors but does correlate with red and white blood cell counts in a german blood donor population. *PLoS One* 10:e0139308. 10.1371/journal.pone.0139308 26445269 PMC4596489

[B52] Nikolin S. Boonstra T. W. Loo C. K. Martin D. (2017). Combined effect of prefrontal transcranial direct current stimulation and a working memory task on heart rate variability. *PLoS One* 12:e0181833. 10.1371/journal.pone.0181833 28771509 PMC5542548

[B53] Nunan D. Sandercock G. R. Brodie D. A. (2010). A quantitative systematic review of normal values for short-term heart rate variability in healthy adults. *Pacing Clin. Electrophysiol.* 33 1407–1417. 10.1111/j.1540-8159.2010.02841.x 20663071

[B54] O’Callaghan N. J. Fenech M. (2011). A quantitative PCR method for measuring absolute telomere length. *Biol. Proced. Online* 13:3. 10.1186/1480-9222-13-3 21369534 PMC3047434

[B55] O’Donovan A. Pantell M. S. Puterman E. Dhabhar F. S. Blackburn E. H. Yaffe K. (2011). Cumulative inflammatory load is associated with short leukocyte telomere length in the health, aging and body composition study. *PLoS One* 6:e19687. 10.1371/journal.pone.0019687 21602933 PMC3094351

[B56] Papaioannou V. Pneumatikos I. Maglaveras N. (2013). Association of heart rate variability and inflammatory response in patients with cardiovascular diseases: Current strengths and limitations. *Front. Physiol.* 4:174. 10.3389/fphys.2013.00174 23847549 PMC3706751

[B57] Perseguini N. M. Verlengia R. Milan J. C. Minatel V. Rehder-Santos P. Takahashi A. C. (2015). Cardiac autonomic modulation, C-reactive protein or telomere length: Which of these variables has greater importance to aging? *Int. J. Cardiol.* 178 79–81. 10.1016/j.ijcard.2014.10.123 25464224

[B58] Pu J. Schmeichel B. J. Demaree H. A. (2010). Cardiac vagal control predicts spontaneous regulation of negative emotional expression and subsequent cognitive performance. *Biol. Psychol.* 84 531–540. 10.1016/j.biopsycho.2009.07.006

[B59] Rao S. G. Jackson J. G. (2016). SASP: Tumor suppressor or promoter? yes! *Trends Cancer* 2 676–687. 10.1016/j.trecan.2016.10.001 28741506

[B60] Rode L. Nordestgaard B. G. Weischer M. Bojesen S. E. (2014). Increased body mass index, elevated c-reactive protein, and short telomere length. *J. Clin. Endocrinol. Metab.* 99:E1671–E1675. 10.1210/jc.2014-1161 24762112

[B61] Rossiello F. Jurk D. Passos J. F. d’Adda di Fagagna F. (2022). Telomere dysfunction in ageing and age-related diseases. *Nat. Cell Biol.* 24 135–147. 10.1038/s41556-022-00842-x 35165420 PMC8985209

[B62] Rufer N. Dragowska W. Thornbury G. Roosnek E. Lansdorp P. M. (1998). Telomere length dynamics in human lymphocyte subpopulations measured by flow cytometry. *Nat. Biotechnol.* 16 743–747. 10.1038/nbt0898-743 9702772

[B63] Schistad E. I. Stubhaug A. Furberg A.-S. Engdahl B. L. Nielsen C. S. (2017). C-reactive protein and cold-pressor tolerance in the general population: The Tromsø study. *Pain* 158 1280–1288. 10.1097/j.pain.0000000000000912 28420008

[B64] Schreck R. Baeuerle P. A. (1994). Assessing oxygen radicals as mediators in activation of inducible eukaryotic transcription factor NF-kappa B. *Methods Enzymol.* 234 151–163. 10.1016/0076-6879(94)34085-4 7808288

[B65] Shaffer F. Ginsberg J. P. (2017). An overview of heart rate variability metrics and norms. *Front. Public Health* 5:258. 10.3389/fpubh.2017.00258 29034226 PMC5624990

[B66] Shahane A. D. LeRoy A. S. Denny B. T. Fagundes C. P. (2020). Connecting cognition, cardiology, and chromosomes: Cognitive reappraisal impacts the relationship between heart rate variability and telomere length in CD8+CD28- cells. *Psychoneuroendocrinology* 112:104517. 10.1016/j.psyneuen.2019.104517 31785500 PMC6935397

[B67] Sproston N. R. Ashworth J. J. (2018). Role of C-Reactive Protein at sites of inflammation and infection. *Front. Immunol.* 9:754. 10.3389/fimmu.2018.00754 29706967 PMC5908901

[B68] Streltsova L. I. Tkacheva Î. V. Plokhova E. V. Akasheva D. U. Strazhesko I. D. Dudinskaya E. (2017). Age-related changes in heart rate variability and their relation with leucocyte telomere length. *Cardiovasc. Ther. Prevent.* 16 54–60. 10.15829/1728-8800-2017-1-54-60

[B69] Sturchio A. Dwivedi A. K. Young C. B. Malm T. Marsili L. Sharma J. S. (2021). High cerebrospinal amyloid-b 42 is associated with normal cognition in individuals with brain amyloidosis. *EClinicalMedicine* 38:100988. 10.1016/j.eclinm.2021.100988 34505023 PMC8413261

[B70] Task Force of the European Society of Cardiology and the North American Society of Pacing and Electrophysiology. (1996). Heart rate variability: Standards of measurement, physiological interpretation and clinical use. *Circulation* 93 1043–1065. 10.1161/01.CIR.93.5.1043 8598068

[B71] Tchkonia T. Zhu Y. van Deursen J. Campisi J. Kirkland J. L. (2013). Cellular senescence and the senescent secretory phenotype: Therapeutic opportunities. *J. Clin. Investig.* 123 966–972. 10.1172/JCI64098 23454759 PMC3582125

[B72] Thayer J. F. Ahs F. Fredrikson M. Sollers J. J. III. Wager T. D. (2012). A meta-analysis of heart rate variability and neuroimaging studies: Implications for heart rate variability as a marker of stress and health. *Neurosci. Biobehav. Rev.* 36 747–756. 10.1016/j.neubiorev.2011.11.009 22178086

[B73] Thayer J. F. Lane R. D. (2000). A model of neurovisceral integration in emotion regulation and dysregulation. *J. Affect. Disord.* 61 201–216. 10.1016/s0165-0327(00)00338-4 11163422

[B74] Tibuakuu M. Kamimura D. Kianoush S. DeFilippis A. P. Al Rifai M. Reynolds L. M. (2017). The association between cigarette smoking and inflammation: The genetic epidemiology network of arteriopathy (GENOA) study. *PLoS One* 12:e0184914. 10.1371/journal.pone.0184914 28922371 PMC5602636

[B75] Tracey K. J. (2002). The inflammatory reflex. *Nature* 420 853–859. 10.1038/nature01321 12490958

[B76] van de Loo A. Mackus M. Kwon O. Krishnakumar I. M. Garssen J. Kraneveld A. D. (2020). The inflammatory response to alcohol consumption and its role in the pathology of alcohol hangover. *J. Clin. Med.* 9:2081. 10.3390/jcm9072081 32630717 PMC7408936

[B77] Victorelli S. Passos J. F. (2017). Telomeres and cell senescence - size matters not. *EBioMedicine* 21 14–20. 10.1016/j.ebiom.2017.03.027 28347656 PMC5514392

[B78] Vizi E. S. (1998). Receptor-mediated local fine-tuning by noradrenergic innervation of neuroendocrine and immune systems. *Ann. N. Y. Acad. Sci.* 851 388–396. 10.1111/j.1749-6632.1998.tb09012.x 9668629

[B79] Wang H. Yu M. Ochani M. Amella C. A. Tanovic M. Susarla S. (2003). Nicotinic acetylcholine receptor alpha7 subunit is an essential regulator of inflammation. *Nature* 421 384–388. 10.1038/nature01339 12508119

[B80] Wang L. Lu Z. Zhao J. Schank M. Cao D. Dang X. (2021). Selective oxidative stress induces dual damage to telomeres and mitochondria in human T cells. *Aging Cell* 20:e13513. 10.1111/acel.13513 34752684 PMC8672791

[B81] Williams D. P. Feeling N. R. Hill L. K. Spangler D. P. Koenig J. Thayer J. F. (2017). Resting heart rate variability, facets of rumination and trait anxiety: Implications for the perseverative cognition hypothesis. *Front. Hum. Neurosci.* 11:520. 10.3389/fnhum.2017.00520 29163100 PMC5671536

[B82] Williams D. P. Koenig J. Carnevali L. Sgoifo A. Jarczok M. N. Sternberg E. M. (2019). Heart rate variability and inflammation: A meta-analysis of human studies. *Brain Behav. Immun*. 80 219–226. 10.1016/j.bbi.2019.03.009 30872091

[B83] Wilson S. J. Woody A. Padin A. C. Lin J. Malarkey W. B. Kiecolt-Glaser J. K. (2019). Loneliness and telomere length: Immune and parasympathetic function in associations with accelerated aging. *Ann. Behav. Med.* 53 541–550. 10.1093/abm/kay064 30107521 PMC6499407

[B84] Wong J. Y. De Vivo I. Lin X. Fang S. C. Christiani D. C. (2014). The relationship between inflammatory biomarkers and telomere length in an occupational prospective cohort study. *PLoS One* 9:e87348. 10.1371/journal.pone.0087348 24475279 PMC3903646

[B85] Wong L. S. Huzen J. de Boer R. A. van Gilst W. H. van Veldhuisen D. J. van der Harst P. (2011). Telomere length of circulating leukocyte subpopulations and buccal cells in patients with ischemic heart failure and their offspring. *PLoS One* 6:e23118. 10.1371/journal.pone.0023118 21876736 PMC3158078

[B86] Wood H. (2018). Dementia: Peripheral inflammation could be a prodromal indicator of dementia. *Nat. Rev. Neurol.* 14:127. 10.1038/nrneurol.2018.8 29377005

[B87] Woody A. Hamilton K. Livitz I. E. Figueroa W. S. Zoccola P. M. (2017). Buccal telomere length and its associations with cortisol, heart rate variability, heart rate, and blood pressure responses to an acute social evaluative stressor in college students. *Stress* 20 249–257. 10.1080/10253890.2017.1328494 28482730

[B88] Zalli A. Carvalho L. A. Lin J. Hamer M. Erusalimsky J. D. Blackburn E. H. (2014). Shorter telomeres with high telomerase activity are associated with raised allostatic load and impoverished psychosocial resources. *Proc. Natl. Acad. Sci. U.S.A.* 111 4519–4524. 10.1073/pnas.1322145111 24616496 PMC3970484

[B89] Zhu X. Han W. Xue W. Zou Y. Xie C. Du J. (2016). The association between telomere length and cancer risk in population studies. *Sci. Rep.* 6:22243. 10.1038/srep22243 26915412 PMC4768100

